# A balance assessment and training protocol to address balance disorders in older and neurologically disabled adults

**DOI:** 10.3389/fresc.2026.1651101

**Published:** 2026-05-29

**Authors:** Joseph Barton, John Sorkin

**Affiliations:** 1 Department of Veterans Affairs, Baltimore VA Medical Center, Baltimore, MD, United States; 2 Department of Neurology, University of Maryland School of Medicine, Baltimore, MD, United States; 3 University of Maryland Center for Vascular Research, University of Maryland School of Medicine, Baltimore, MD, United States

**Keywords:** balance assessment, balance rehabilitation training, fear of falling (FOF), human balance, human balance control

## Abstract

Falls are by far the leading cause of accidental injury and death in older adults. Despite its importance, much is still unknown about the manner in which balance is controlled, and how it is compromised by age, disease, and injury. Diagnostic instruments that assess balance and fall risk have proven to be of limited utility, as have therapeutic interventions. The Balance Assessment and Training Protocol (BATP) was developed to address these shortcomings. It incorporates separate Assessment and Training Modules that establish subjects' Limit of Balance (LoB), compute a target trajectory based on it that requires subjects to remain at their LoB in order to track it, and then assess or train balance performance in this task. The BATP continuously challenges subjects to perform reaching tasks at the limits of their balance, and increases these limits as subjects demonstrate improved performance. The goal of the BATP is to quantitatively assess and improve at-risk individuals' ability to maintain balance when disturbed by volitional movements of the body and its parts—an important class of balance disturbances integral to many activities of daily living that can precipitate falls. The BATP focuses on performance at and just beyond the LoB, unlike most such tests and training protocols that do not challenge subjects in this way. We hypothesize that expanding this boundary, as the BATP is designed to do, will improve balance and make individuals more resistant to falls in everyday activities.

## Introduction

Impaired balance contributes to falls and functional impairment in older (65+ years old) and disabled (e.g., stroke) populations; and falls are by far the leading cause of accidental injury and death in this population segment (1). In 2023, this segment (comprising 18% of the US population) experienced 3.9 million fall-related injuries. 43,000 died from falls, and fall-related medical expenses totaled $94 billion. Overall, fall-related injuries constitute 56% of all unintentional injuries leading to death in older adults (1).

### Extant balance assessment instruments

A variety of clinical diagnostic instruments have been developed to assess balance and fall risk (2), but have proven to be of limited usefulness in various ways (3). These include the use of subjective ordinal scales which do not lend themselves to deeper and more extensive quantitative analyses; lack of hierarchical structure and inclusion of redundant items that artificially inflate scores; applicability to only select populations; or relevance for only a narrow range of subject performance, exhibiting floor or ceiling effects when performance falls outside that range. The Berg Balance Scale (4), Physical Performance Test (5), and Performance-Oriented Mobility Assessment (6) are examples of such instruments. As people age many develop Fear of Falling (**FoF**). FoF is the most prevalent fear of older adults, affecting over 50% of this population (7). Forty percent of older adults restrict movements and activities as a result of FoF (8). This leads to reduced physical conditioning and ability to perform Instrumental Activities of Daily Living (**IADLs**) (9), reduced social interaction, and ironically, increased FoF and falls (10). FoF is most commonly measured by subjective self-assessments such as Falls Efficacy Scale (**FES**) (11) and the Activities-Specific Balance Confidence (**ABC**) Scale (12), in which individuals rate their perceived ability to avoid a fall while performing IADLs, or their confidence that they can perform IADLs without falling (7). There are to our knowledge no objective, quantitative measures of FoF. Despite this, Landers (13) found measures such as these to be better predictors of falls than clinical measures of balance in common use.

Several dynamic balance instruments have also been developed. These include the Functional Reach test (14), Y-Balance and Star Excursion Tests (15), and the Limits of Stability (**LoS**) test (16). These tests involve bending at the waist and reaching as far as possible with the dominant hand in various directions, standing on one foot and reaching as far as possible with the other in various directions, or standing in the standard position and leaning as far as possible in various directions. In each the reaching or leaning distance is used as a measure of balance capability. While these instruments are an improvement over those described above, they are not without their own limitations. The procedures they employ closely correspond to a classical Psychophysical instrument, the Method of Adjustment (17), and thus share its vulnerabilities. The Method of Adjustment is self-administered, which can introduce bias into the measurements. It is also vulnerable to habituation errors (limiting reach/lean based on habits formed from previous efforts) and anticipation errors (limiting reach/lean prematurely in anticipation of becoming overextended).

All of these instruments were intended to be quick, easily administered tests that could be performed in a physician's office or clinic with minimal equipment. They were designed to provide a consistent and repeatable “score” of balance capability that could be used to compare (at least subjectively) capability across subjects, or changes in a single subject's capability over time. With few exceptions, they were not intended to diagnose the causes of diminished balance capability, or to permit deeper, quantitative analyses of balance function, and their ability to do so is quite limited. Though they largely accomplish their intended purpose, they are often not sufficient to meet the practice needs of rehabilitation professionals (18), or provide only limited information beyond that obtained through direct clinical observation (19, 20). There is presently no “gold standard” with which to quantitatively assess balance performance and fall risk, and in practice people with poor balance or at high risk for falling are typically identified tautologically as individuals who have fallen in the recent past (under conditions in which a person unaffected by age or disability would not have fallen).

### Extant balance training regimens

Several different training regimens have been developed to address balance deficits in older people. Multi-Modal Balance Training (**MMBI**) is perhaps the most widely employed, and typically consists of a variety of exercises designed to improve balance, flexibility, and endurance (21). Single leg stance, stretching, and treadmill walking, respectively, are examples of each form of exercise. Tai Chi training, in which individuals repetitively perform a variety of movements emphasizing single- and double-leg weight-bearing stances and postural alignment has also been employed (22). A recent Cochrane Report (23) found that MMBI produced a statistically significant reduction in fall rate of 29%. Tai Chi training produced a 28% reduction but this was not statistically significant due to the heterogeneity of the studies sampled. Resistance training (24) failed to produce a significant decrease in fall rate. The ∼30% reductions in fall rates reported by the Cochrane meta-analysis are substantial, given the numbers of people, falls, and moneys involved. However, this still leaves 70% of falls unaddressed. One limitation of these interventions is that they do not isolate and challenge specific balance performance deficits sufficiently to ameliorate them (25). A second limitation is that the tasks performed in these interventions do not sufficiently simulate the real-life conditions under which people fall. Because of these it has been estimated that the maximum reduction in fall rate that we can expect from such interventions is 30%–40% (26).

### The mechanics of balance

**Figure 1:**
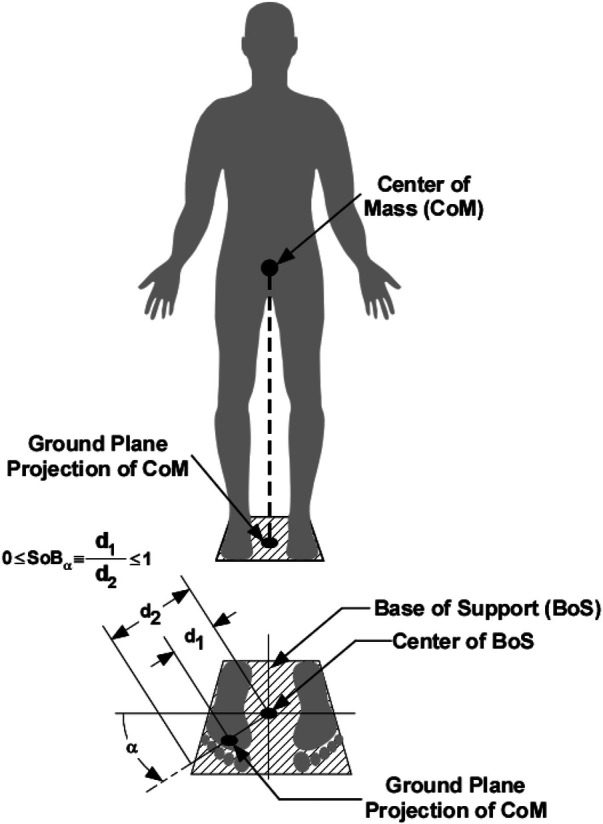
The mechanical requirement for static balance is that the projection of the CoM onto the ground plane lie within the perimeter of the BoS.

The mechanical requirement for any static structure to remain upright is that the Ground Plane Projection (**GPP**) of its Center of Mass (**CoM**) lie within the perimeter of its Base of Support (**BoS**). For human standing balance, the BoS is the polygon that just encloses the surfaces of contact of the two feet (27, 28) (Figure 1). The requirement for the stability of a static structure does not hold, however, in the case of dynamic stance, e.g., when a standing individual leans towards the perimeter of their BoS. Hof et al. (29) and Patton et al. (30, 31) have investigated the mechanical requirement for stability, or Limit of Stability (LoS), for dynamic stance,. Hof, et al. represented a standing individual in the standard position swaying towards the perimeter of their BoS at some CoM velocity *v* as a two-link inverted pendulum. They determined that upright stance can be maintained as long as the Ground Plane Projection of the Center of Mass (**GPP-CoM**) remains a distance $v\sqrt{l/g}$ from the perimeter of the BoS, where *l* is the distance from the ankle center of rotation to the CoM and *g* is gravitational acceleration. Ankle torque was not explicitly included in their derivation, but its presence is implied since according to the inverted pendulum model it is the only means by which the locations of the GPP-CoM and Center of Pressure (**CoP**) can be controlled. (CoP was included in the derivation but does not appear in the final result because for this limiting case it lies on the perimeter of the BoS.) The result also assumes that for the given CoM velocity, sufficient ankle torque can be generated quickly enough to maintain the required distance from the perimeter of the BoS. For a young healthy individual in quiet sway such an assumption is reasonable. For the subject populations and movement tasks our assessment tasks are designed for, however, neither this assumption nor the inverted pendulum model would be valid. A fuller and more comprehensive *systems view* of balance function (which subsumes the concept of LoS) is necessary to properly evaluate it under these more general conditions.

The ***Systems View of Balance Function*** is based upon the Internal Model (**IM**) paradigm (32, 33), which posits that balance is controlled by numerous sensorimotor and biomechanical elements organized into an adaptive feedback control loop. Within this loop highly adaptable networks of neurons “model” the kinematics and dynamics of the body and its parts, and the forces and constraints imposed by the external environment. These networks estimate current and future “states” of balance; accounting for an individual's sensorimotor and musculoskeletal condition and capabilities, the body's momentum (mass × velocity) in approaching the perimeter of the BoS, and any relevant features of the external environment. If an out-of-balance condition is detected they activate the musculature to counter it (34). This feedback control loop also performs an adaptive function. As commands are issued to the musculature, the body's response is predicted and then compared to its actual response as monitored by the sensory system. Based on this, any errant neural processes involved in these tasks are modified to improve their accuracy.

To characterize and quantify this loop's function we define the State of Balance (**SoB**) as the distance from the center of the BoS to the GPP-CoM (d_1_ in Figure 1); divided by the distance from the center of the BoS to the perimeter of the BoS (d_2_). For stable motion SoB must lie within the range 0 ≤ SoB ≤ 1. While this range holds for movement in any direction (*α* in Figure 1), the same value of SoB in various directions will involve different values of d_1_ and d_2_, given the asymmetry of the BoS. If a standing individual moves their body in a way that will cause the GPP-CoM to move beyond the perimeter of the BoS, they must either curtail its movement to prevent this or step to expand their BoS. Either action must be initiated far enough in advance of reaching the perimeter of the BoS (i.e., for some SoB < 1) to allow sufficient time and space to complete it and prevent a fall. The Limit of Balance (**LoB**) is that value of SoB < 1 at which this curtailment or step must be initiated. An Out-of-Balance (**OoB**) condition exists when SoB ≥ LoB. SoB, LoB and OoB are perceived quantities estimated by the neural processes within the balance control loop, based upon signals provided by the different sensory modalities involved in balance. The cumulative effects of age, disease, and injury cause the body, including the elements of the balance control loop itself, to deteriorate over time (35, 36). In the case of movement and balance, manifestations of this deterioration include decreased joint ranges of motion, slower and weaker muscle response dynamics, slower and less accurate sensory and motor control dynamics, increased neural processing and transmission delays, and increased neural transmission “noise”. The ability to both detect and respond to OoB conditions is negatively affected, and in compensation the balance control loop reduces its estimate of LoB.

While this depiction of dynamic balance is consistent with that of Hof and Patton et al., it greatly expands upon it by incorporating all of the factors affecting balance control, including LoS. Furthermore, it emphasizes the role that perception plays in estimating quantities such as SoB, LoB, and OoB, and provides a means to account for these percepts' inherently uncertain nature. It can also account for the effects of delays and noise around the control loop, and changes in the ability to control balance over both the short and long term. In view of this we expect LoB to impose a greater restriction on dynamic stance than LoS.

As we will describe, LoB will be determined experimentally as part of the Balance Assessment and Training Protocol's (**BATP's**) Assessment and Training procedures. In a separate, independent study, we are developing a mathematical model of the balance control loop we have just described to analytically determine, among other quantities, subjects' LoB in performing the BATP's tracking task. This model will incorporate sensory and higher level neural processing and transmission delays, the effects of neural transmission noise, and muscle response dynamics to provide a comprehensive simulation of performance in the task. The tracking task requires subjects to bend at the waist and reach as far to the left or right as they can when the target moves to either side, and to fully extend their bodies and reach up and forward as far as they can when the target moves to those positions. Such movements could not be accurately captured by Hof et al.'s two-link inverted pendulum model, and we instead plan to represent the body with a 15-segment biomechanical model.

### The balanced reach test—precursor to the BATP

**Figure 2:**
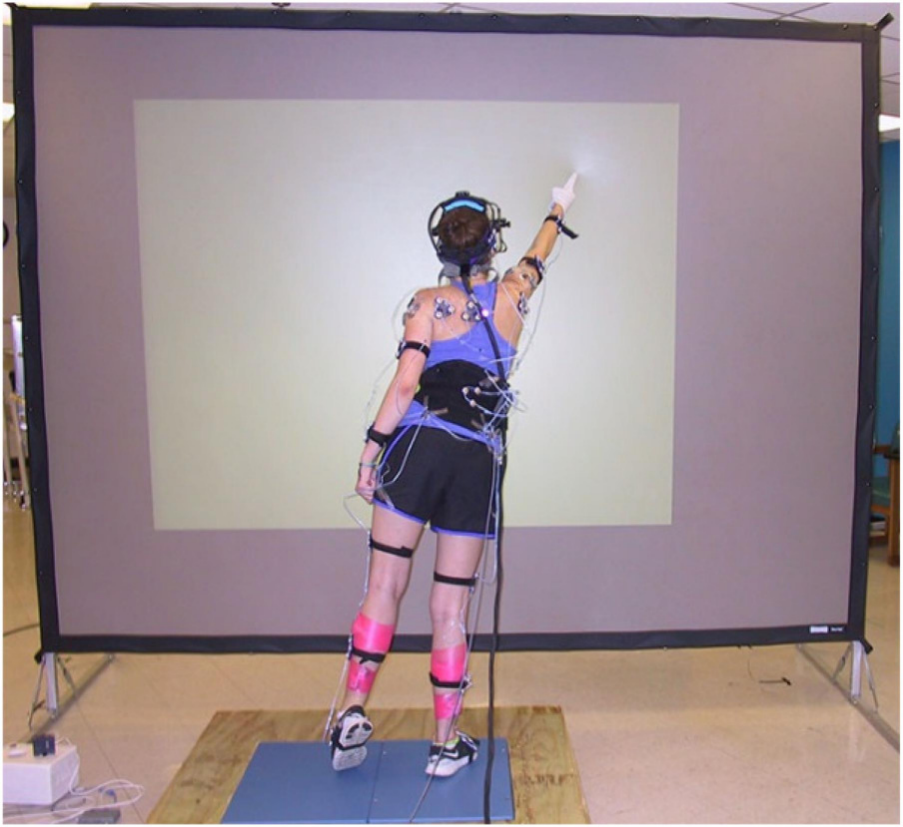
During the balanced reach test (BRT), subjects stood on force platforms, fixed gaze and pointed to the projected disk as it moved around the screen. The locations of four landmarks on each foot were used to compute the perimeter of the BoS at each sampling instant. This computation was robust enough to accurately represent the perimeter should the subject shift their stance or momentarily raise up on the fronts of one or both feet (as shown in the figure).

Prior to the BATP we developed the Balanced Reach Test (**BRT**) to address the limitations of extant balance assessment instruments. In it (Figure 2), subjects stood with each foot supported by a separate 6 degree-of-freedom force plate. The feet were positioned directly beneath the center of rotation of each foot's respective shoulder. A 7′ 6″ × 10′ reverse-projection screen was located approximately 1–1½ arm lengths in front of the subject. An image of a disk was projected onto the screen from the rear and the subject pointed with the dominant hand index finger to its center as it moved about the screen, lightly grazing the screen with their fingertip. Subjects were instructed not to step, but were allowed to raise up onto the front of their feet (Figure 2). Body movements undertaken to track the disk are integral to many daily activities and are sufficient to precipitate a fall (37, 38). The Right-Left (**RL**) (x) and Superior-Inferior (**SI**) (y) positions of the disk at time t were determined by a sum of 14 sine functions, given by

$$x_{d}(t)=\sum_{i\;=\;1}^{14}\frac{1}{2\pi f_{i}}\sin\;(2\pi f_{i}\;t),\;y_{d}(t)=\sum_{i\;=\;1}^{14}\frac{1}{2\pi f_{i}}\sin\;(2\pi f_{i}\;t+\phi_{i}).$$

**Figure 3:**
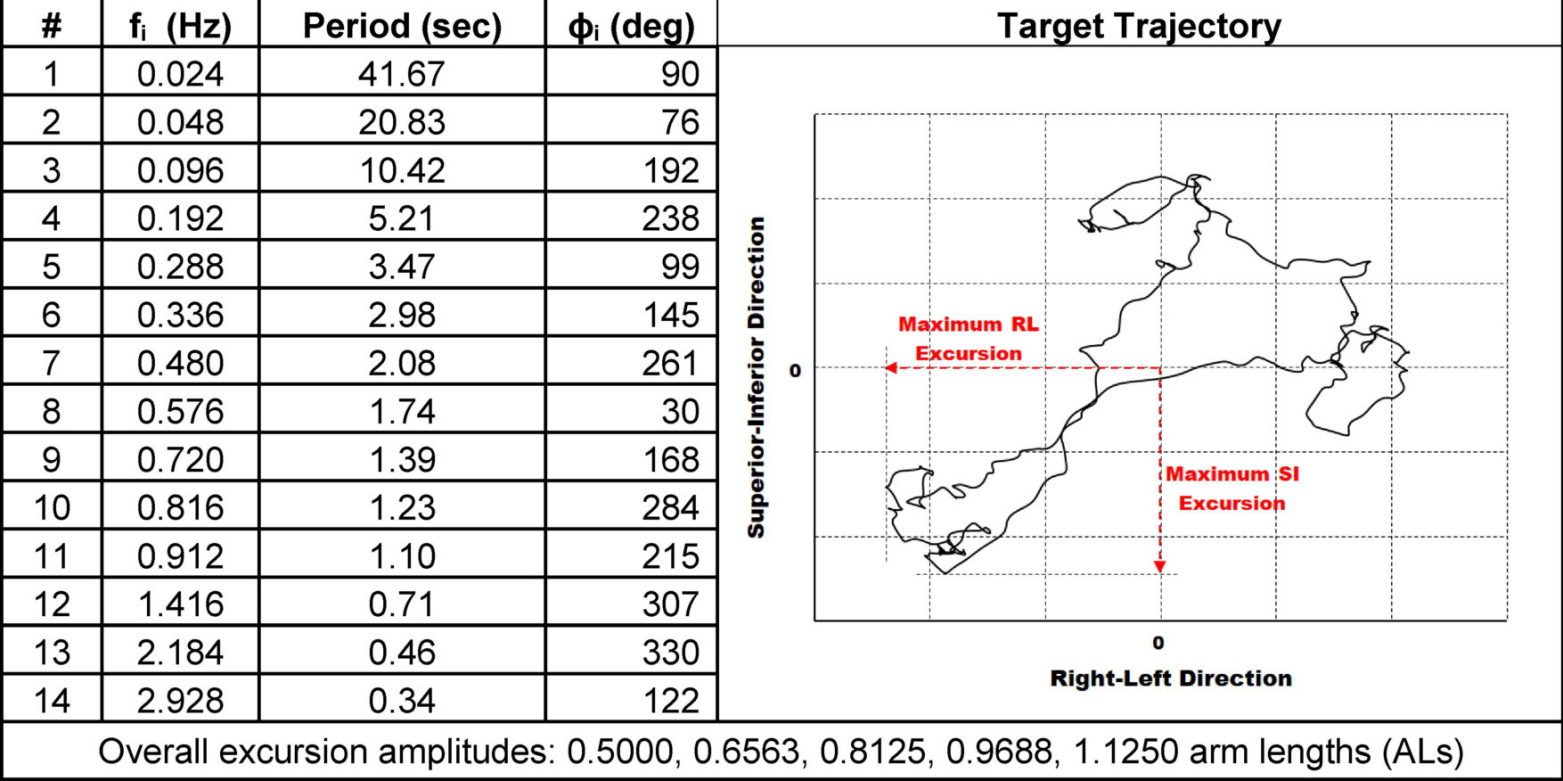
Target motion parameters and trajectory.

Such a stimulus appears random to the observer. We used 10 frequencies approximately uniformly distributed across a range (0.05 ≤ *f_i_* ≤ 3.0 Hz) (Figure 3) that can be visually tracked by healthy subjects (39), and four additional frequencies in the vicinity of 1 Hz where we found tracking gain changed most rapidly. Amplitudes of individual sinusoids were scaled by the inverse of their respective frequencies so that each sinusoid had the same peak velocity. A randomly selected phase shift within the range 0 ≤ *ϕ*_I_ ≤ 360 (Figure 3) was applied to each term of the SI sum-of-sines, *y_d_*(*t*), so that the vertical component of the disk's motion did not appear correlated with the horizontal component. The frequencies were also chosen to be integer multiples of the lowest, or fundamental frequency (0.024 Hz) in the range. As a result all of the higher frequency components of the trajectory complete a cycle at the same instant as the fundamental frequency (∼41.67 s). This, then, is the period of the entire trajectory, shown in Figure 3. The tracking task involved tracking the target for two cycles of this trajectory. This choice of frequencies and duration simplified spectral analyses of target and subject motion data because it eliminated spectral leakage that occurs when a measured signal starts or ends in mid-cycle.

At the beginning of each experiment the subject's Arm Length (**AL**) was measured as the distance from the tip of the pointing index finger to the acromion process. Five multiples of arm length evenly distributed along a logarathmic scale between 0.5 to 1.125 (Figure 3, bottom) were then computed. Once the disk's overall trajectory was computed, the maximum excursions of the RL and SI components were determined (Figure 3). The RL and SI components were then divided by their respective maximums, scaling each component to a maximum value of one. For each test condition, or trial, this scaled trajectory was then multiplied by the appropriate multiple of arm length. Increasing the overall excursion amplitude of the target trajectory increased task difficulty in that it caused the target trajectory (and the subject's CoM trajectory) to expand outward and move closer to the limits of the subject's Base of Support (BoS), requiring the subject to assume increasingly less stable body poses with respect to the BoS in order to accurately track the target. Task difficulty was further increased because the target's velocity increased, since for sinusoidal motion velocity is directly proportional to amplitude. Subjects completed five ∼83 s trials corresponding to each of the five different overall excursion amplitudes. As subjects performed the tracking task the movements of each of the 15 body segments making up their body, the tip of the tracking finger, and the tips of the toes, heels, and outer sides of each foot (defining the BoS) were recorded by a motion capture system. Ground reaction forces, moments, and CoP for each foot were recorded by the two force plates.

**Figure 4:**
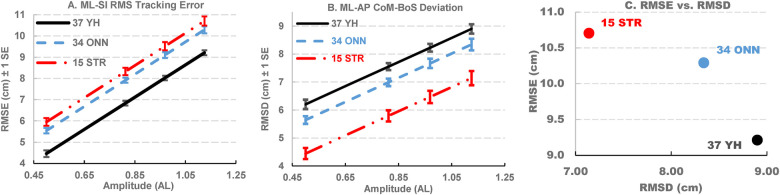
YH adults exhibited the smallest tracking error **(A)**, and the largest CoM-BoS deviation **(B)** in the BRT, followed by ONN and STR subjects. The inverse relation between tracking accuracy and balance stability is demonstrated in **(C)**.

We assessed performance in the BRT using measures of tracking accuracy and balance stability. Tracking accuracy is defined by the Root Mean Squared (**RMS**) Error (**RMSE**) between the disk center and the tracking fingertip. Balance stability is defined by the RMS Deviation (**RMSD**) between whole body GPP-CoM and the center of the BoS. Lower values of RMSE indicate better tracking performance; higher values of RMSD indicate more confidence in balance capability and a greater willingness to move and remain closer to the limits of the BoS. We tested 37 Young Healthy (**YH**) adults, 34 Older Non-Neurologically disabled (**ONN**) adults (adults free from the effects of Stroke, TBI, Parkinson's disease, etc.), and 15 older stroke (**STR**) survivors, age matched to the ONN. We developed a linear mixed effects model (quantified by RMSE and RMSD) to assess the effects of different levels of task difficulty (increasing disk excursion amplitude) in the different groups. We found that for all three groups RMSE and RMSD increased with increasing disk excursion amplitude (Figures 4A,B). Moreover, tracking error was higher with more severe deficits in the balance control system, in that RMSE_STR_ > RMSE_ONN_ > RMSE_YH_ for all amplitudes (Figure 4A). Similarly, willingness to challenge balance was lower as the severity of balance system deficits increased, in that RMSD_STR_ < RMSD_ONN_ < RMSD_YH_ for all amplitudes (Figure 4B). All of these results are statistically significant (*p* < 0.05). Figure 4C shows RMSE as a function of RMSD for each population group at the greatest excursion amplitude (1.125 arm lengths). The YH group challenged their balance the most (greatest RMSD) and achieved the lowest RMSE. Conversely, the STR group challenged balance the least (lowest RMSD) and achieved the greatest RMSE. The performance of the ONN group fell between these two. The plot thus exhibits an inverse relation; i.e., a tradeoff, between RMSD and RMSE. Table 1 provides group averaged RMSE and RMSD for each excursion amplitude and population group, based upon the measurement data that the linear mixed effects model was derived from. We also found evidence of a training effect (not shown) in a subset of ONN and STR subjects who repeated the test 48 h later. The results of the BRT experiment have been reported previously [58, 59].

**Table 1:** Group averaged RMSE, RMSD, and ellipse/BoS ratios based on experimental data used to derive the linear mixed effects model summarized in Figure 4. All values in cm. Standard errors are shown in parentheses.

Amplitude (AL)	37 YHS		34 ONN		15 STR	
	RMSE	RMSD	RMSE	RMSD	RMSE	RMSD
0.5	4.58 (0.12)	6.05 (0.24)	5.47 (0.16)	5.72 (0.19)	5.83 (0.31)	4.61 (0.26)
0.6563	5.72 (0.09)	6.81 (0.18)	6.71 (0.12)	6.30 (0.15)	7.12 (0.24)	5.12 (0.20)
0.8125	6.87 (0.07)	7.56 (0.14)	7.96 (0.14)	6.88 (0.17)	8.41 (0.29)	5.62 (0.25)
0.9688	8.01 (0.07)	8.32 (0.15)	9.20 (0.19)	7.46 (0.24)	9.70 (0.42)	6.13 (0.37)
1.125	9.16 (0.10)	9.07 (0.19)	10.45 (0.27)	8.04 (0.33)	10.99 (0.58)	6.64 (0.51)
Ellipse-BOS Ratio (Std Err)	0.51 (0.03)		0.29 (0.02)		0.17 (0.02)	

**Figure 5:**
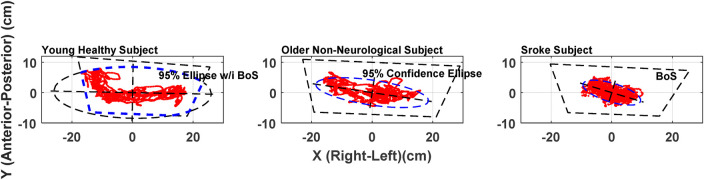
CoP trajectories (red dots) for a representative YH, ONN and STR subject performing the BRT. YH subjects used most of their available BoS area during the task (51%), followed in turn by ONN (29%) and STR subjects (17%).

The relative performance of the three subject groups was further demonstrated by comparing the motion of their Centers of Pressure (CoPs) during the BRT. Figure 5 shows the CoP trajectory (red dots) for a representative subject from each group performing the BRT at an overall target excursion amplitude of 1.1250 arm lengths. Also shown is the 95% Confidence Ellipse around the CoP trajectory (95% of CoP points fall within this ellipse) and each subject's base of support (BoS, defined in Figure 1). As evidenced by the sizes of the 95% Confidence Ellipses relative to the sizes of the BoS's, the YH subject moved about the most within the BoS, moved the farthest from its center (the most stable state of balance), and also moved closest to its boundaries. This was followed in turn by the ONN and the STR subject. This indicates that the YH subject was most willing to challenge their balance in order to accurately track the target while the ONN and STR subjects were progressively less willing to do so. To quantify this we computed the ratio of the area of the confidence ellipse falling within the BoS (outlined in blue in Figure 5) to the area of the BoS. The ratios are presented in Table 1, indicating that YH subjects used 51% of their BoS, followed in turn by ONN (29%) and STR subjects (17%). These differences are also statistically significant (*p* ≤ 0.02).

These results demonstrate that the BRT provides an objective, quantitative assessment of balance performance with sufficient sensitivity to distinguish between the three subject groups. It contrasts with the clinical measures described earlier and addresses their shortcomings in several important ways. The BRT comprises the dual task of **a)** reaching and pointing to a moving target while **b)** maintaining balance in response to the disturbances caused by these reaching and pointing movements. The random nature of the target's motion prevents the subject from anticipating the next required body movement to successfully track the target, and the requirement to track the target accurately focuses attention on tracking the target and makes balance maintenance a secondary focus. The task was designed to simulate an activity (reaching in one's environment to acquire and manipulate objects) that is fundamental to many IADLs, and that can precipitate a fall, in that the body and body segment movements undertaken to track the target are sufficient to upset balance (37, 38). The BRT's outcome measures are free of the shortcomings associated with the clinical instruments described earlier, and the recorded data provides a rich data set for deeper and more extensive quantitative analyses.

Further analysis of the measurement data revealed that differences between the performance of the subject groups were most pronounced when the target was farthest away from them; and reduced when the target was closer in (Figure 3). The BRT, then, derived most of its discriminatory power from performance data taken at the extremes of the target's trajectory, and the subjects' reach. This finding could perhaps have been predicted (at least with the aid of hindsight). When individuals perform an unchallenging movement task there is great latitude in the manner in which they can execute it, given the degree to which sensorimotor and musculoskeletal elements can substitute and compensate for one another (40). As a result, the performance of different members of the same subject group, and even that of the same individual from one execution of the task to the next, can be quite variable. Assessments made under such conditions may not fully or reliably characterize and distinguish an individual's or group's full capabilities in the task. When individuals are given a task requiring them to perform at the limits of their capabilities, however, their ability to adapt and compensate is minimized. Their performance becomes more repeatable and stereotypical, and representative of their subject group (40). As a result, measurement resolution, repeatability, and the ability to discriminate between groups with different performance capabilities should increase. This was a primary consideration in the design of the BATP's tracking task, as we describe next.

## Balance assessment and training protocol (BATP)—successor of the BRT

The BATP, which follows on from the BRT and is the subject of this article, modifies and expands upon the BRT to take advantage of the earlier study's findings. It consists of separate Assessment and Training Modules, the latter to exploit the BRT's observed training effect and investigate its effectiveness as a balance training regimen. Each module employs a tracking task similar to that of the BRT. It has been modified, though, to force subjects to remain at the limit of their reach (and therefore balance) in order to track the target accurately. At the beginning of each assessment or training session, subjects' maximal reach in various directions on the projection screen will first be located. Their Limit of Reach (**LoR**) in a particular direction corresponds to their LoB in that direction. These points will then be used to compute a trajectory that is always at the subject's LoR, forcing them to remain at their LoB in order to track it. Subjects will participate in multiple assessment and training sessions and at each their LoR/LoB will be re-assessed and the tracking trajectory recomputed. This will ensure that subjects continue to perform the task at their current LoB throughout the study. We hypothesize that the sensitivity and resolution (and thus discriminatory power) of the BATP's assessments will be greatly improved vs. the BRT and extant clinical measures of balance. We also hypothesize that as BATP training progresses, subjects' balance performance will improve (i.e., LoR and LoB will expand outward and FoF will decrease). Furthermore, we believe this will lead to lasting and significant improvements in balance capability, beyond what is currently achieved with extant balance training regimens. Increasing the difficulty of the tracking task as balance performance and FoF improve is an example of performance accomplishment: the mastery of increasingly difficult tasks. It is a key element of Bandura's Self-Efficacy Theory (41). Such modification is particularly important for the maintenance of good function in individuals at risk of functional decline (42).

### BATP materials, equipment, setup, & operation

**Figure 6:**
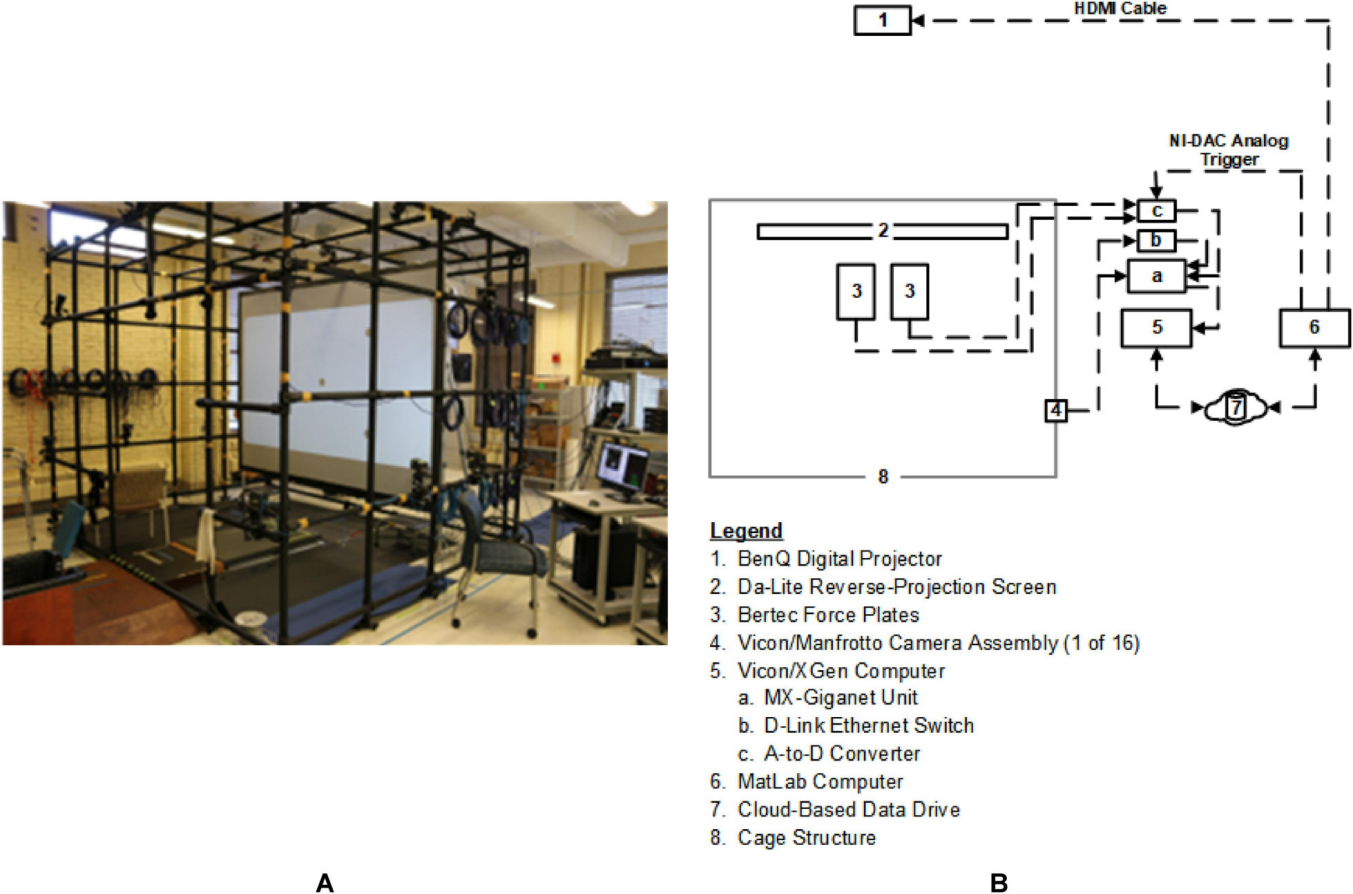
**(A)** vicon “Cage” structure. **(B)** Component Layout & Interconnections.

The materials and equipment employed to implement the BATP are listed in Table 2. The test facility, which was recently constructed, is shown in Figure 6A, and a diagrammatic sketch showing how its components are interconnected is shown in Figure 6B. Construction of the test facility began with that of the 12 ½′ L × 10′ W × 10′ H Cage Structure that encloses the motion capture space, and on which 16 Vicon cameras are mounted. It is constructed with 1½″ Schedule 40 aluminum pipe and connecting fittings (Hollaender Corp.). The fittings connecting the horizontal pipes of the cage allow them to be moved up or down along the vertical pipes. This allows the motion capture cameras to be mounted at any position surrounding the capture space (see below). To prevent reflection from the pipe and fittings, they have been painted with flat-black spray paint (Krylon Corp.). The Cage's design and listings of its components are described in greater detail in the Supplementary Appendix.

**Table 2:** Materials and equipment employed by the BATP.

#	Equipment
1	Vicon Motion Capture System
	− 16 Bonita B10 Infrared Cameras with Manfrotto 804RC2 Basic Pan Tilt Head w/ Quick Lock Manfrotto 200PL Quick Release Plate Manfrotto 035 Super Clamp − MX-Giganet Unit − D-Link DGS-1510-28P Ethernet Switch − 24 Channel A-to-D Converter
2	Two Bertec FP4060-10-2000 Force Plates
3	Dell Precision T7920 Tower (Windows 11)
	− Vicon Nexus 2 Software Application − Motion Monitor xGen Software Application
4	MatLab Computer (Windows 10)
	− National Instruments PCI-6255 Data Acquisition Card − MatLab Software Application w/ 3rd Party PsychToolbox-3
5	Da-Lite 6′ × 10′ Reverse-Projection Screen
6	BenQ SP 890 High Resolution Digital Projector
7	HTC VIVE Pro Eye Office Virtual Reality System (to replace Items 5 & 6)
8	12 ½′L **×** 10′ W **×** 10′ H Cage Structure (Supplementary Appendix)

As with the BRT, performance in the BATP will be evaluated using measurements of whole body and body segment motion and the forces and moments applied to the ground by each foot, as subjects perform the balanced reach tasks included in the Assessment and Training modules. Whole body and body segment motion will be measured using a Vicon 3D motion capture system consisting of 16 Bonita B10 infrared cameras connected directly to a Vicon MX-Giganet Unit or a D-Link DGS-1510-28P Ethernet Switch connected to the MX-Giganet Unit. The cameras will track whole body and body segment motion by recording the positions of reflective infrared markers attached to the subject's body segments at each sampling instant, as described below. Applied forces and moments will be measured by two Bertec FP4060-10-2000 force plates (one supporting each foot) connected to a 24 channel A-to-D converter, which also connects to the MX-Giganet Unit.

During experiments motion capture and force plate data will be collected at 100 samples per second and transmitted via an ethernet connection to the MX-Giganet Unit, and in turn to a Windows 11 Dell Precision T7920 Tower computer running the Vicon Nexus 2 software application. The Nexus application will initiate and terminate data collection and ensure that all data are in synchrony (i.e., sampled at the same instants in time). At the beginning of each session a calibration will be performed that establishes a Global Coordinate System (**GCS**); to which each camera's and force plate's Local Coordinate System (**LCS**) will be referenced (see Methods). In order for subjects' motions to be captured in their entirety throughout each measurement run it is necessary for each infrared reflective marker to be in view of at least two Bonita cameras at all times. To ensure this, the cameras have been mounted on the cage structure as described earlier.

**Figure 7:**
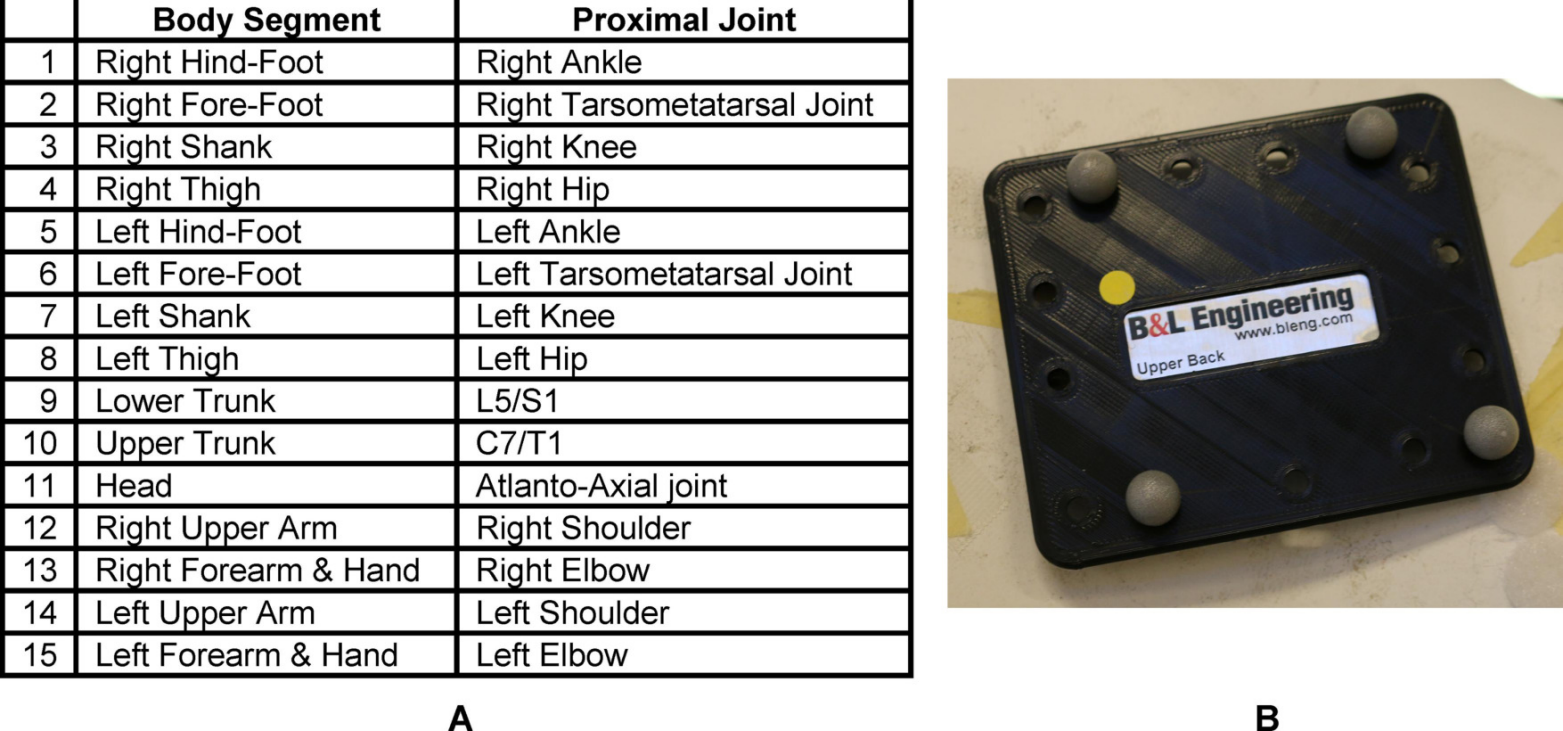
**(A)** body segments and associated proximal joints. **(B)** Rigid Body Cluster.

Each body segment is assumed to act as a rigid body, which exhibits both linear and rotational motion. To capture both components of motion a “rigid body cluster” (B&L Engineering) (Figure 7B) will be firmly attached to it using an elastic band. Each body segment has a unique cluster geometry, which is defined in the Vicon Nexus application. Three or four infrared markers are attached to the cluster's rigid base plate and do not move with respect to one another. The arrangement can then used to establish an LCS fixed to each body segment. The origin of this LCS is located at the body segment's CoM, and its *x*-axis is aligned to the body segment's long axis. Each body segment begins and ends at a joint. At the beginning of each assessment or training session a second, “subject calibration” will be performed in which the LCS for each body segment is established. The locations of these end points (and the joints they correspond to) will then be established in LCS coordinates, and referred to the GCS established previously. These processes are described more fully below (see Methods). Once established, the body segment and joint data can be combined into an open kinematic chain providing a digital, biomechanical representation of the entire body. Such a representation will be created for each subject, for each assessment and training session. Figure 7A lists the body segments and end points/joints defined for the BATP.

The subject calibration process by which body segment and whole body representations are created will be carried out by the Motion Monitor xGen software application (Innovative Sports Training Inc.), which is also resident on the Dell Precision T7920 Tower. To accomplish this the xGen application interfaces with and takes control of the Nexus application, such that the computer operator interacts solely with the xGen application. Once this second calibration is complete the operator conducts the assessment or training session through the xGen application. The assessment and training sessions each consist of a number of balance tasks for which data is collected. After each balance task is completed all measurement data are transmitted directly from the Vicon Nexus application to the xGen application, which saves the results to file. The xGen application is capable of creating a wide variety of different “reports” using this measurement data and generating text files of the results that can then be accessed by other applications for more extensive follow-on analyses. For these studies the xGen application will be used to report the linear motion (position/angle vs. time) of each body segment's CoM as well as that of the whole body, and the angular motion of each body segment about its CoM. It will also report the forces and moments applied by each foot as measured by the Bertec force plates.

The balance tasks employed by the BATP involve reaching and pointing to a target as it moves about the projection screen, as shown in Figure 2. The trajectory that the target follows for each task will be computed using a custom MatLab program, which resides on a second computer running in tandem with the Dell Precision T7920 Tower. We refer to this Windows 10 computer as the “MatLab Computer”. The MatLab application employs PsychToolbox-3, a third party toolbox, to create an animation of the target disk moving along this trajectory in real time, which is projected onto a Da-Lite 6′ × 10′ Reverse-Projection Screen via a BenQ SP 890 High Resolution Digital Projector.

A National Instruments PCI-6255 Data Acquisition Card (**NI-DAC**) has been installed on the MatLab Computer which can read and transmit analog and digital signals. As an assessment or training session progresses the MatLab program will specify the next balance task to be performed and at the operator's command will issue an electronic trigger via the NI-DAC to the 24 channel A-to-D converter, which will be passed to the xGen application on the T7920 Tower. This will simultaneously initiate the target motion and data collection while just prior to this the operator will inform the subject to track the target. When the target completes its motion the xGen application will terminate data collection. Once a balance task is completed reports of select measurement data will be generated by the xGen application and transferred to the MatLab Computer via a cloud-based data drive. The custom MatLab application will use this data to compute the target trajectory or other parameters used in the next phase of the assessment or training session, as described below.

## Methods

### Sample size

Using the BRT we were able to distinguish between three groups, YH, ONN, STR with a maximum of 34 subjects in the more heterogeneous ONN and STR groups. As described earlier (Introduction), we expect that the BATP, by testing and training subjects at their LOB, will produce data that are more accurate and precise than the BRT. Given that the training and testing tasks of the BATP will be the same as the BRT (other than both being performed at subjects' LoB) and that the data will be gathered with higher accuracy and greater precision, we anticipate that much like the data from the BRT, having 34 subjects in each group will allow us to distinguish between the groups.

### Eligibility criteria

**Figure 8:**
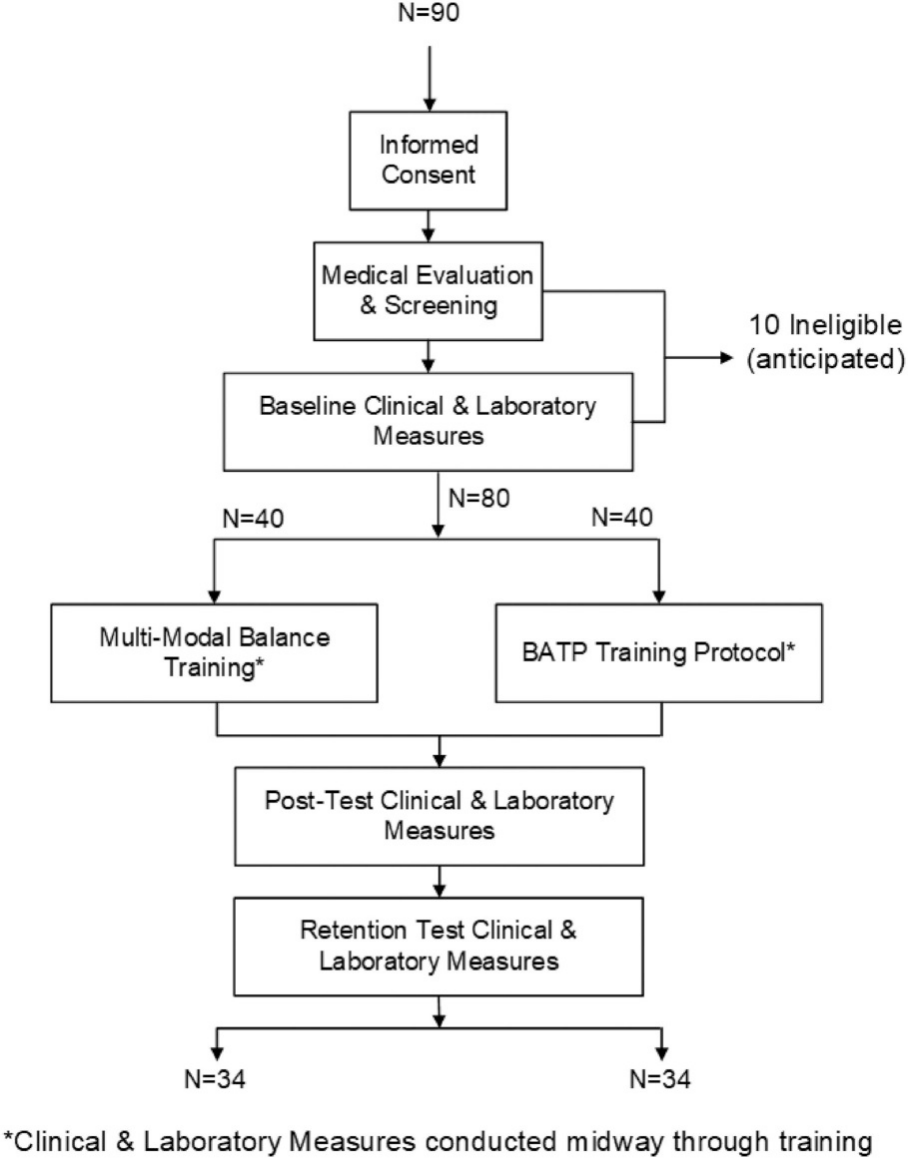
Movement of test subjects through study.

We will screen candidates other than YH candidates for medical and cardiopulmonary conditions that preclude testing or exercise. If any are found we will refer them with their permission to their care providers to address any medical needs. Once conditions are treated and medical clearance documented, subjects will be eligible to screen again for participation. We are currently comparing the effects of two training regimens, the BATP regimen and the MMBI regimen. Movement of test subjects through this study is shown in Figure 8.

### Inclusion criteria

1. Men or women age 60–85
2. High Fall Risk: Fallen twice or more in the past year
3. Adequate language and neurocognitive function to participate in testing and training
4. Able to give adequate informed consent
5. Ability to perform BATP: able to perform the balanced reach task without assistive devices
6. Ability to perform MMBI: Able to rise from a chair unaided and walk 10 meters without human assistance
7. Vision adequate to see a 1 inch diameter black disk against a white background at 3 feet.

### Exclusion criteria

Clinical history of

1. Unstable angina
2. Recent myocardial infarction (<3 month) or hemodynamically significant congestive heart failure (NYHA II) or valvular dysfunction
3. Peripheral arterial occlusive disease with claudication
4. Hip fractures, other lower extremity large bone fractures occurring within the past year, other serious musculoskeletal injuries, upper or lower body orthopedic issues, and/or major chronic pain that would prevent the subject from performing the balanced reach task
5. Pulmonary failure
6. Body Mass Index (BMI) > 40
7. Active vertigo
8. Symptomatic orthostatic hypotension
9. Poorly controlled hypertension (>190/105) on at least two separate occasions
10. Poorly controlled type 1 or 2 diabetes (HbA1c > 10)
11. Recent hospitalization for severe disease or surgery (<3 month)
12. Excessive daily alcohol consumption (>3 oz. liquor; >12 oz. wine; or >36 oz. beer) or illicit drug abuse
13. Untreated major clinical depression or dementia
14. ONN evaluations: Neurological disease or injury such as stroke, TBI, Parkinson's disease, etc.
15. STR evaluations: Neurological disease or injury other than stroke
16. Vestibular disorders of sufficient severity to prevent the subject from performing the balanced reach task
17. Any other condition (e.g., extreme frailty) that would preclude safe completion of the BATP or MMBI.

### Pre-Assessment/training session setup

Prior to an Assessment or Training session the Vicon system (including the force plates) will be calibrated according to the Vicon Users' Guide and Reference Guide (43, 44). This involves 1) “zeroing” the force plates; 2) masking spurious infrared reflections appearing in the motion capture space; 3) calibrating and synchronizing the cameras; and 4) establishing the GCS origin and orientation. An array of target disks will then be displayed on the projection screen, as computed by the MatLab program using the two-dimensional coordinate system of the projection screen (pixels), and their positions recorded by the Vicon system using a stylus. Using both the computed screen coordinates and the measured Vicon coordinates, the MatLab program will locate the plane of the projection screen in the Vicon GCS and compute a transformation allowing us to represent any computed position of the target disk in Vicon coordinates and visa versa. Rigid body clusters will then be fixed to the subject's body segments and pointing finger and the subject will be “calibrated” using the Motion Monitor xGen application. To verify that these steps have been completed correctly, a second array of target disks will be projected onto the screen by the MatLab program and the subject will be instructed to point to the center of each one in turn with their pointing fingertip, lightly making contact with the screen. The MatLab program will then compare the measured position of the pointing fingertip with the computed position of the target disk (transformed from screen coordinates to Vicon coordinates) to ensure they coincide. This procedure allows us to represent disk motion, motion capture, and force plate measurements in the same coordinate system so that we can accurately compare and correlate subject responses to target motion.

### BATP assessment sessions

The BATP Assessment Sessions consist of four separate measurement tasks: **a)** the administration of accepted Clinical Measures of Balance, **b)** the Step Response Task, **c)** the Limit of Reach/Balance Task, and **d)** the Balance Assessment Task. A BATP Assessment Session will be conducted at four points during training. Prior to training at baseline (**B**), mid-way through training (**MT**), post training (i.e., immediately after training) (**PT**), and six weeks after training is complete to assess the training regimen's retention effect (**R**). For all balance tasks subjects will be harnessed to an overhead cable and “spotted” by laboratory staff to guard against falling. The spotter will also observe subjects throughout all of the Assessment and Training sessions to ensure they are fully engaged in the tasks and performing them to the best of their ability (i.e., at their LoR/LoB). They will monitor subjects for fatigue, pain, or other discomfort that would negatively affect performance, and call rest breaks when warranted. In addition to preventing injury in the event of a fall, the spotter will also record the circumstances of the fall for later evaluation. Since measurement data will also be recorded throughout all sessions, a quantitative record of the fall will be available for more extensive analyses.

While ***Clinical Measures of Balance*** have proven to be of limited usefulness, as discussed earlier, we include them as part of our assessments because they provide familiar and common points of reference for clinicians and balance researchers. Their inclusion also affords us the opportunity to compare them to and correlate them with the results of our balanced reach assessments. The following widely accepted measures will be administered to subjects:

1. Four Square Step Test (**FSST**) (45)
2. Five Times Sit to Stand (**FTSTS**) (46)
3. Multi-Directional Reach Test (**MDRT**) (47)
4. Mini Balance Evaluation Systems Test (**Mini-BESTest**) (48)
5. Disabilities of the Arm, Shoulder, and Head (**DASH**) Test (stroke subjects only) (49)
6. Berg Balance Scale (**BBS**) (stroke subjects only) (50)
7. Falls Efficacy Scale (**FES**) (11), which assesses FoF
8. Activities-Specific Balance Confidence (**ABC**) Scale (12), which assesses FoF
9. Postural Sway Test (**PST**) (Eyes Open and Eyes Closed) (51)
10. While not a clinical measure, we also maintain a record of falls experienced by each subject, as well as the conditions in which they fell, during and throughout the first year following training.

***The Step Response Task*** evaluates how quickly subjects are able to assess, plan, and execute body movements in response to unexpected, fast-changing stimuli. In it the target disk is projected onto the projection screen directly anterior to the pointing finger's shoulder center of rotation. This point is located during the Pre-Assessment/Training Session Setup using the Motion Monitor XGen application with the subject assuming the standard anatomical position. The subject is then instructed to point to the target and after a short delay the target unexpectedly moves radially outward in one of eight directions (0°, 45°, 90°, 135°, 180°, 225°, 270°, and 315° counterclockwise from right-side horizontal). In each instance the target moves ∼0.7 Arm Lengths in a straight line in 0.5 s. The order of presentation is random. The subject is instructed to track the target with their fingertip as quickly and accurately as they can without stepping. This task is not intended to challenge subjects' balance, and the target's travel remains well within their LoR/LoB.

**Figure 9:**
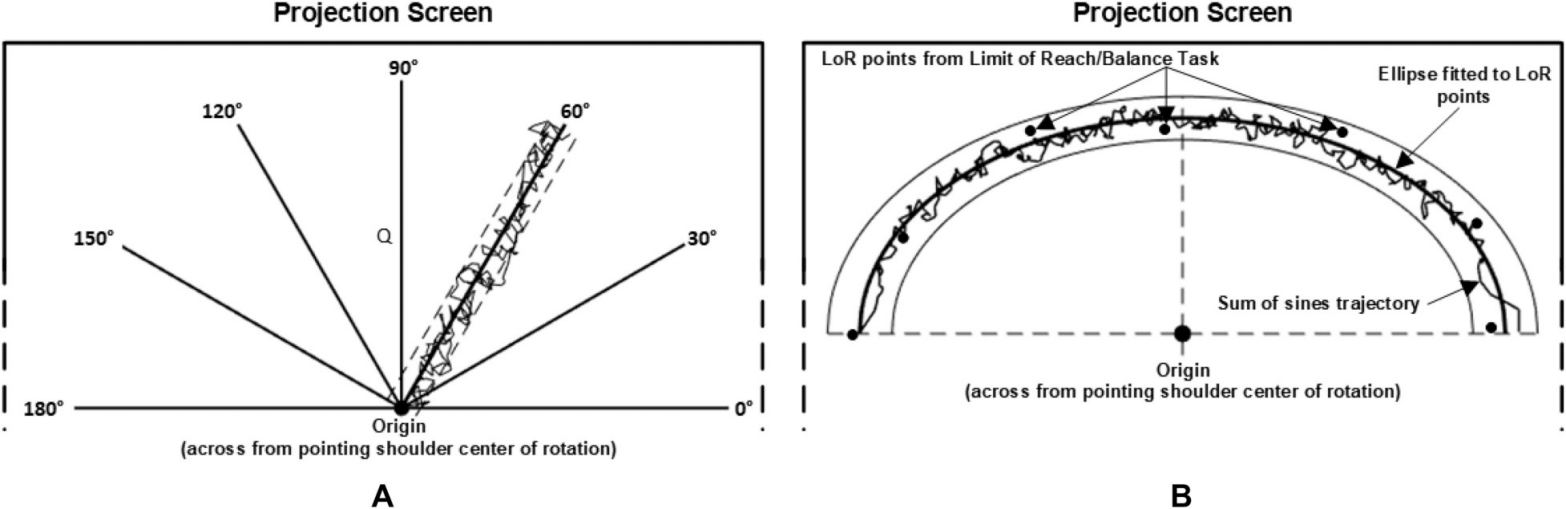
**(A)** disk motion directions corresponding to LoB. **(B)** Construction of elliptical region of disk motion.

***The Limit of Reach/Balance Task*** establishes LoR/LoB corresponding to seven disk motion directions (0°, 30°, 60°, 90°, 120°, 150°, and 180° from the horizontal) defined by lines three arm lengths long extending radially from an origin located on the projection screen directly anterior to the pointing finger's shoulder center of rotation (Figure 9A). Seven directions were chosen as the protocol was being developed because this number provided a well-defined ellipse (see below) that would accurately capture a subject's full range of maximal reach in the tracking task while not being overly burdensome to complete. The order of presentation is random. After a short delay the target unexpectedly moves radially outward in one of the seven directions. Target motion is programmed to move in a random-like fashion along one of the specified directional lines (Figure 9A). The random element of this motion is according to a sum-of-sines function having the same frequencies as those used in the BRT, but scaled to a smaller overall amplitude to remain within a six-inch-wide band centered along the specified directional line. The subject tracks the target without stepping, using their dominant hand index finger. The random nature of the target's motion prevents the subject from anticipating the next required body movement to successfully track the target. This focuses attention on tracking the target and makes balance maintenance a secondary focus. The target moves away from the subject and eventually beyond their reach (and LoB). The subject tracks the target to this point and then holds their position as the target moves beyond it, maintaining this pose until the target reaches the end of its travel. When the task is complete the MatLab program compares the trajectories of the target and pointing fingertip for each motion direction and identifies the points at which these trajectories deviate significantly. These positions are taken to coincide with the subject's LoR/LoB in these directions. Once these points are identified the MatLab program fits an ellipse to them using *lsqnonlin*, its nonlinear least squares subroutine (Figure 9B).

For the ***Balance Assessment Task*** the MatLab program computes an Assessment Trajectory in which the target moves unpredictably along the upper half of the LoR/LoB Ellipse in a random fashion (Figure 9B). The random element of this motion is again according to a sum-of-sines function having the same frequencies as those used in the BRT, but scaled to a smaller overall amplitude to remain within a six-inch-wide band centered along the fitted ellipse. Comparison with Figure 3 shows how this trajectory differs from that used in the BRT. As with the BRT, the Assessment trajectory completes two cycles of the computed trajectory. It completes the first cycle moving from the right-most end of the trajectory to the left-most end, and the second cycle moving back from the left-most end to the right-most end. The total duration of the target motion is ∼83 s Subjects track this trajectory two times.

### BATP training sessions

The BATP Training Sessions begin by administering the Limit of Reach/Balance Task and computing the unpredictable target disk trajectory based upon it. This Training Trajectory is computed in the same way and for the same duration as the Assessment Trajectory described previously. We have found that older and disabled subjects are only able to track the target for this amount of time before becoming overly fatigued. A single presentation of the Training Trajectory constitutes one training bout. The test administrator specifies the number of bouts the subject engages in, with the ultimate goal of completing 43 (approximately one hour of total training), to be consistent with extant balance training regimens. Subjects are allowed to rest between bouts whenever requested. In practice it has been necessary to begin with a lesser number of training bouts and gradually increase them to the desired number over time. Subjects train three times a week for six weeks (18 Training Sessions), to be consistent with extant balance training regimens and the MMBI training group. As training progresses and the subject's performance improves, the BATP will present increasingly difficult (i.e, expanded) target trajectories based upon the increased LoR/LoBs and corresponding overall target excursion amplitudes as measured during LoR/LoB task at the beginning of each training session.

In parallel with BATP training, a second cohort of subjects will participate in MMBI training, also consisting of 18 one-hour training sessions over six weeks. To compare the effects of the two regimens, these subjects will also undergo the BATP Assessment Session at the beginning of training, midway through training, at the end of training, and six weeks after training is complete.

### Measurement data

In addition to the data obtained from the Clinical Measures of Balance, the following measurement data will be recorded at a rate of 100 samples/sec during each assessment task and training session:

1. The motion (position vs. time) of the target disk;
2. The motion of each of 15 body segments (Table 2), the tip of the tracking finger, and the tips of the toes, heels, and outer sides of each foot (defining the BoS) (Vicon Motion Capture System);
3. Ground reaction forces, moments, and CoP for each foot and for both feet combined (dual Bertec force plates).

The measurement data will be recorded throughout the tracking period for all of the Assessment tasks and Training bouts. It will then be used to compute the BATP's outcome measures:

4. RMSE;
5. RMSD;
6. SoB as a function of time, and average SoB;
7. LoB;
8. LoB Ellipse and BoS parameters used in the construction of Figure 5.
9.LoR Ellipse parameters used in the construction of Figure 9B.

While the analyses described below can be performed using any of these measures, we will, unless otherwise specified, perform them using LoB, which we designate our *primary* outcome measure. Certain of these analyses will also be conducted using several Clinical Measures of Balance that we will have collected along with the BATP measures. These are the Four Square Step Test (**FSST**), the Five Times Sit to Stand test (**FTSTS**), the Multi-Directional Reach Test (**MDRT**), the Falls Efficacy Scale (**FES**), and the Postural Sway Test (**PST**). All of these measures except the FES are obtained on a ratio scale and can be used in mathematical calculations (52). The FES is obtained on an ordinal scale, however, and cannot be used this way. Special analytical methods are required when using it, as we explain below.

### Analyses of measurement data

There are many different analyses that can be performed with the extensive and rich data sets generated by the BATP. Here we limit our discussion to those that directly address the primary goals of our current study:

1a. To assess the ability of the BATP's Assessment Module to provide quantitative evaluations of balance performance with sufficient sensitivity to distinguish between population groups with different kinds of balance deficits.
1b. To compare the sensitivity of the BATP's Assessment Module's primary outcome measures with those of the BRT.
2a. To assess the training efficacy of BATP's Training Module by comparing subjects' balance performance during and after training to that of a control group training according to the MMBI.
2b. To evaluate the degree to which subjects' performance as measured by the BATP's outcome measures correlate with performance measured by the Clinical Measures of Balance administered at the same time points during and after training.
3. To compare the rate of falls through one-year post training in subjects who receive BATP training to falls in subjects who receive MMBI training.

### Analyses methods (by aim)

1a. ONN and STR subjects will be assessed at four time points, baseline (**B**), midway through training (**MT**), immediately after training (**PT**), and six-weeks after training (**R)**. YH subjects will be assessed only at the first time point, B. At the first time point the mean LoB's of all three groups will be compared. At the second through fourth time points the ONN and STR LoB's will be compared to one another. If the means are significantly different from one another (*p* = 0.05) at each time point the BATP's Assessment Module will be judged to have sufficient sensitivity to distinguish between population groups with different kinds of balance deficits.
1b. The BRT and BATP have two outcome measures in common, RMSE and RMSD, which will be measured in each of our three groups. The BRT measures taken at the highest tracking excursion amplitude (1.125 AL) will compared to the corresponding BATP measures taken at Baseline. The BATP will be considered superior to the BRT if the 95% confidence intervals around the mean RMSE and mean RMSD of the BATP are significantly smaller (*p* = 0.05) than the confidence intervals obtained using the BRT's data.
2a We will assess and compare the training efficacy of ONN and STR subjects using measures of LoB taken at four time points: baseline (**B**) mid-training (**MT**), post-training (**PT**), and retention six-weeks after the last training session (**R**). We will use repeated measures ANOVA (SAS *proc mixed*) in which the dependent variable will be the within person change from one time period to the next, i.e., MT-B, PT-MT, R-PT. The model will be adjusted for group (BATP, MMBI); time period (early training [B to MT], late training [MT-PT], overall training (B-PT], retention [PT to R]); and a time period × group interaction. If we find a significant period × group effect we will use *post-hoc* comparisons to determine the time points that show significant differences. We will use linear contrasts to **1)** compare the efficacy of the BATP to that of the MMBI by comparing the within group changes during training; and **2)** compare the ability of the two interventions to engender a long-lasting improvement in balance by comparing the percentage change during retention. We will use AICC, a modification of Akaike's information criteria (53, 54), to determine the covariance structure (e.g., unstructured, compound symmetry, first order auto-regressive) that best accounts for the serial autocorrelation of repeated observations obtained from the same subject.
  Prior to accepting the results from our analyses, we will assure that data conform to the assumptions of the analyses (e.g., normal distribution of residuals), and that no datum has excessive influence or leverage (e.g., Cook's D). We will use multiple imputation to impute follow-up data for subjects missing values. Multiple imputation (55) yields unbiased estimates if the pattern of missing data is missing completely at random. Each primary hypotheses will be tested independently of the others. Armitage (56) holds that a small number of *a priori* primary endpoints can be taken at their face value and no correction for multiple comparisons is necessary. We will therefore deem a two-tailed test *p* < 0.05 as indicating statistical significance. We expect the BATP to be more effective in improving functional balance than MMBI, and that this improvement will last six weeks after training. We further expect the improvement to be clinically significant.
  We will also assess training effectiveness using the select Clinical Measures of Balance we listed above, which are also obtained at each of the four time points. Changes in performance based on the FSST, MDRT, FTSTS, and PST will be analyzed in the same manner as described above. For the FES, a subjective assessment will be employed to evaluate performance changes based on it.
2b. We will use Pearson's *ρ* to assess correlations between LoB and the FSST, MDRT, FTSTS, and PST; and Spearman's *ρ* to assess correlations between LoB and the FES.
3. We will use Poisson regression [with General Estimating Equations of Liang and Zeiger (57) to account for serial autocorrelation of repeated measures from the same subject] to compare the rate of falls between the BATP and MMBI training groups

### Anticipated results

We expect the BATP's performance measures to exhibit greater resolution, and thus greater sensitivity, than those of its predecessor, the BRT, as well as the clinical measures of balance. As a result the BATP will distinguish differences in performance across subjects and subject groups, and changes in the performance of the same subjects and subject groups over time as a result of training, to a greater degree than either the BRT or the clinical measures of balance. We also expect BATP Training to be more effective in improving functional balance than the MMBI, as measured by the BATP performance measures, the clinical measures of balance, and fall rates; that this improvement will last six weeks after training, and that the differences will be clinically significant.

We expect the BATP measures to correlate with the clinical measures, but to vary in a “smoother” and more continuous manner, and exhibit greater range and sensitivity. We expect these correlations to be statistically significant, providing evidence that the BATP-based balance measures have clinical validity. Since the Multifunctional Reach Test and Falls Efficacy Scale have also been shown to correlate with fall risk, statistically significant correlations of BATP measures with these measures will provide evidence that LoB is indicative of fall risk. Finally, a statistically significant correlation between LoB and the FES will provide evidence that LoB can serve as a quantitative measure of FoF.

## Discussion

Our current study addresses balance deficits in older high fall risk and stroke populations. The BATP is capable of addressing balance disorders in a wide range of other affected population groups. These include both younger and older individuals with physiological deficits affecting balance, such as arthritis, sciatica, diabetic and non-diabetic peripheral neuropathy, and HIV; and individuals with other neurological disorders such as traumatic brain injury, Parkinson's Disease, and dementia.

Earlier we limited our discussion of the analyses that we planned to perform to those that would directly address the primary goals of our current study, but noted that many other analyses were possible given the extensive data sets we were compiling. We plan to replicate two analyses that we previously performed using data from the BATP's precursor, the Balanced Reach Test. The first will be an inverse dynamics analysis to compute the forces and torques applied at each of the joints during the course of the BATP tracking task, using kinematic and ground reaction force data from either the Assessment or Training sessions. The earlier analysis using BRT data (58) demonstrated that the joints act in a different but highly coordinated manner to accomplish the tracking task—with individual joints responding congruently to different portions of the target disk's frequency spectrum. In the second analysis (59) we performed detailed spectral analyses of group-representative response data for each of the BRT's five overall excursion amplitudes. We derived empirical and analytical transfer functions between the motion of the disk and that of the tracking finger and CoM, computed tracking and CoM responses to a step input, and showed how RMSE and RMSD varied as functions of disk frequency. We expect the results of these follow-on analyses using BATP data to exhibit greater resolution and ability to discriminate between the performance of different subject groups.

Earlier we mentioned that we are independently developing a Control System Model of standing dynamic Balance (CSMB)—a mathematical model of the balance system's adaptive feedback control loop—to simulate and predict performance in the BATP's tracking task. The CSMB is a mechanistic/statistical model, composed of quantitative “sub-models” of the primary sensorimotor and biomechanical components comprising the balance system's adaptive feedback control loop. The sensory and biomechanical components are developed with reference to the known physiology and function of their real-life counterparts. The predictive, motor control, and adaptive neural processes are modeled according to the Internal Model paradigm (33, 60). A subject-specific instantiation of the CSMB is created by fitting its component sub-models to data from a series of subject-specific performance assessments (separate from the BATP) to specify their constant parameters. These assessments are valuable in their own right and can also be used directly to assess a subject's balance function vis-à-vis comparisons to the performance characteristics of “normal” young healthy individuals. The remaining parameters are then quantified by fitting the entire model to performance in the BATP tracking task. Once the CSMB has been quantified in this manner it can be used to show how departures of these performance characteristics from normal affect observed performance in the BATP tracking task. Assessments can include the manner in which sensory integration processes integrate sensory information when one or more modalities is impaired; how lower body joint activations change to compensate for individual joint deficits; and how balance performance is affected by increased neural noise and processing/transmission delays associated with one or more of the balance system's neural elements. The CSMB can also be used to simulate a particular subject's performance in the tracking task and used to compute outcome measures such as RMSE, RMSD, SoB, and LoB for comparison with their measured counterparts. Upon successful completion of the CSMB we plan to integrate it into the BATP's analytical protocols, and make it part of our assessment repertoire. To our knowledge this constitutes the first effort to develop a comprehensive, quantitative model of human balance that describes performance in fully three dimensional movement scenarios that simulate conditions under which people fall. It incorporates each of the human balance system's primary elements into a neurophysiologically accurate structure and simulates their function in a manner consistent with observed performance.

The BATP is unable to fully assess or train performance in the Anterior-Posterior (**AP**) direction due to the constraining presence of the projection screen. To remove this constraint we are developing a Virtual Reality (**VR**) version of the BATP (**VR-BATP**). It will employ VR eyewear to replace the projection screen. In it, a virtual target sphere will be presented to subjects in fully three dimensional virtual space, using an HTC VIVE Pro Eye Office Virtual Reality System in place of the projection screen and projector. The “workflow” required to present a target moving in virtual 3D space via VR eyewear differs from that used to present the 2D target moving around the projector screen. In the latter, the MatLab application that controls the assessment or training session computes the trajectory (position vs. time) that the target will move along during the next movement task, and then displays an animation of the target moving along this trajectory on the projection screen via a series of calls to functions provided by the third party toolbox PsychToolbox-3. This operation is seamless because PsychToolbox-3's functions are all executed within the MatLab-programming environment. The animation is initiated by a keypress, which also transmits an electronic trigger to the BATP's measurement systems, initiating data collection at the same instant. In the case of VR, the target trajectory must be transmitted to a separate 3D computer graphics game engine, which renders the animation and presents it via the VR eyewear. We have selected Unreal Engine 5.7 for this purpose and are currently developing the workflow and script necessary to duplicate the previous workflow's functionality.

## Data Availability

The original contributions presented in the study are included in the article/Supplementary Material, further inquiries can be directed to the corresponding author.
