# Supplementary material for: A balance assessment and training protocol to address balance disorders in older and neurologically disabled adults
Source: Front Rehabil Sci. 2026 May 29;7:1651101. doi: 10.3389/fresc.2026.1651101 (PMC13260060; doi:10.3389/fresc.2026.1651101)
Supplement: Supplementary file 1 [file Supplementaryfile1.docx]

**Supplementary Material: Design and Components for BATP “Cage”**

Fig. A1 shows the cage layout, and Table A1 lists all of the pipe rails and fittings used in its construction. Hollaender catalog entries for the fittings and the MSC catalog entry for the Leg Dollies are shown in Fig. A2. All of these components are manufactured by Hollaender, Inc., except the Leg Dollies, which are manufactured by Vestil; and all were purchased from MSC Industrial Supply Co. (<https://www.mscdirect.com/>). In Fig. A1 all of the vertical rails and the horizontal rails that form the corners of the cage are fixed and do not move. They are connected by in-line fittings. These components are all shown in black in the figure. The rails shown in green are able to slide along the fixed rails and are connected by offset fittings. With this arrangement a rail can be placed anywhere on the surface of the cage, allowing the Vicon cameras which are mounted on them to capture a subject’s movements from any angle. This is necessary because in the reaching task subjects will bend and twist to maintain contact with the target disk, and individual infrared marker clusters can become obscured for important phases of the movement task. Once in place all of the rails are locked by tightening the set screws mounted in the fittings. This results in a very rigid, secure structure. The Leg Dollies are placed under each vertical rail and enable the cage to be moved about. Given its weight, the cage does not move readily. Any small movements that it might experience (e.g., resulting from building vibrations) do not compromise measurements, as such displacements are accounted for when the cameras are calibrated at the beginning of each session.

Each Vicon camera is mounted on a Manfrotto Pan Tilt Head, using a Manfrotto Quick Release Plate. This assembly is then clamped to a rail in any desired position using a Manfrotto Super Clamp. These items are shown in Fig. A3. They were purchased from B&H Photo (<https://www.bhphotovideo.com/>).


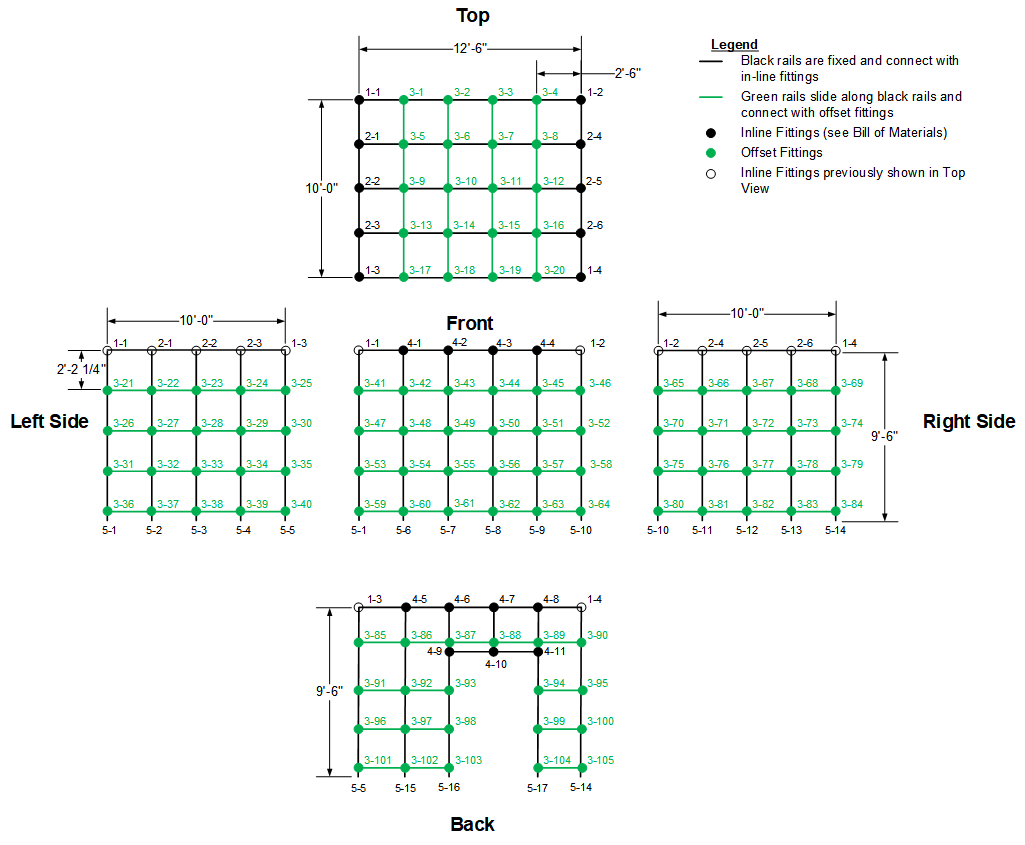


**Fig. A1: BATP Cage Layout**

**Table A1: BATP Cage Bill of Materials**

| **#** | **Hollaender Fittings** | **Item No.** | **Quantity** | **Unit Wgt (lbs)** | **Wgt (lbs)** |
| --- | --- | --- | --- | --- | --- |
| 1 | # 9 Side Outlet Elbow | 8120 | 4 | 0.68 | 2.72 |
| 2 | # 11 Side Outlet Tee | 8140 | 6 | 0.70 | 4.20 |
| 3 | # 12 Short Barrel Cross | 8160 | 105 | 0.81 | 85.05 |
| 4 | # 5 Tee | 8040 | 11 | 0.62 | 6.82 |
| 5 | #62 Plug | 8610 | 17 | 0.12 | 2.04 |
| 6 | Set Screw JS 600 Plated 5/16 - 18 x 5/1 | 80312 | 50 | 0.02 | 1.00 |
| 7 | VESTIL Leg Dolly | 94558517 | 17 | N/A | N/A |
|  | **Total** |  | 143 |  | 100.8 |

| **#** | **Rail Sections** | **Length** | **Length** | **Quantity** | **Total Length (ft)** | **Wgt (lbs)** |
| --- | --- | --- | --- | --- | --- | --- |
| 1 | 1-1/2" SCH 40 Mill Aluminum Pipe | 12.500 | 12' 6" | 5 | 62.5 | 60.0 |
| 2 | 1-1/2" SCH 40 Mill Aluminum Pipe | 12.708 | 12' 8-1/2" | 5 | 63.5 | 61.0 |
| 3 | 1-1/2" SCH 40 Mill Aluminum Pipe | 9.500 | 9' 6" | 17 | 161.5 | 155.0 |
| 4 | 1-1/2" SCH 40 Mill Aluminum Pipe | 10.000 | 10' 0" | 2 | 20.0 | 19.2 |
| 5 | 1-1/2" SCH 40 Mill Aluminum Pipe | 10.208 | 10' 2-1/2" | 12 | 122.5 | 117.6 |
| 6 | 1-1/2" SCH 40 Mill Aluminum Pipe | 5.000 | 5' 0" | 1 | 5.0 | 4.8 |
| 7 | 1-1/2" SCH 40 Mill Aluminum Pipe | 5.208 | 5' 2-1/2" | 3 | 15.6 | 15.0 |
| 8 | 1-1/2" SCH 40 Mill Aluminum Pipe | 2.500 | 2' 6" | 1 | 2.5 | 2.4 |
| 9 | 1-1/2" SCH 40 Mill Aluminum Pipe | 2.708 | 2' 8-1/2" | 3 | 8.1 | 7.8 |
|  | **Total** |  |  | 49 | 461.3 | 442.8 |


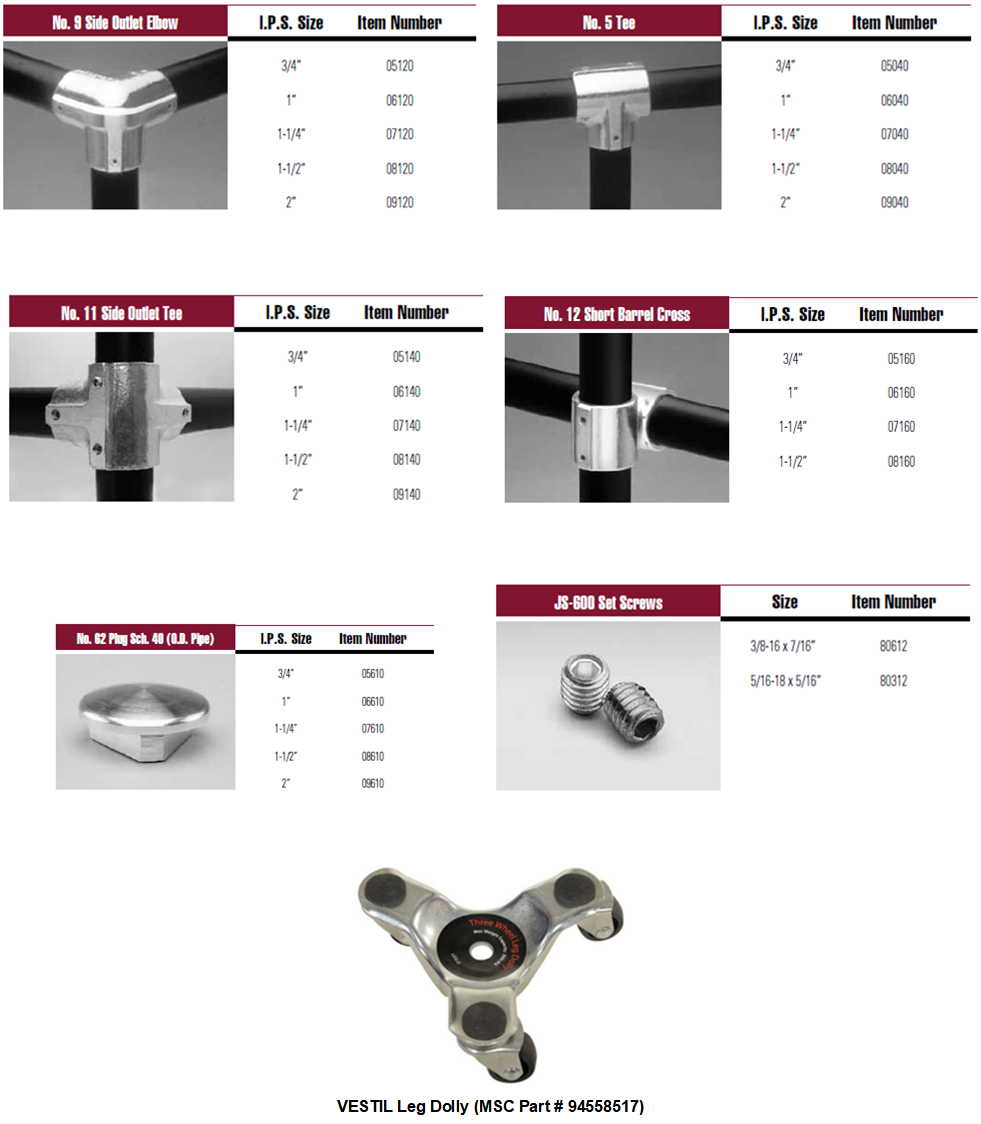


**Fig. A2: Hollaender Catalog Entries (except Leg Dolly, which is produced by Vestil)**


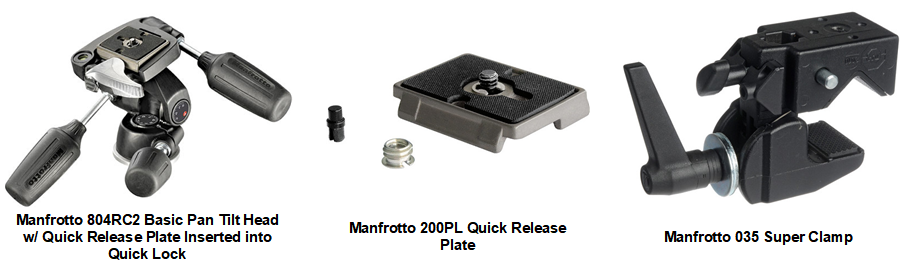


**Fig. A3: Manfrotto Camera Mounting Accessories**
